# Centering strengths of traditional knowledge and culturally sensitive interventions: Strategizing provision of prediabetes wellness interventions for indigenous peoples and ethnic minorities in the United States and across Southeast Asia

**DOI:** 10.1111/1753-0407.13330

**Published:** 2022-11-28

**Authors:** Sheena Ramazanu, Emily Ang, Intan Azura Mokhtar, Jamie Cahoon, Sandra del Pino, Susana Gomez

**Affiliations:** ^1^ National University of Singapore, Alice Lee Centre for Nursing Studies, Yong Loo Lin School of Medicine National University of Singapore, Level 3, Clinical Research Centre Singapore Singapore; ^2^ Alice Lee Centre for Nursing Studies, Yong Loo Lin School of Medicine National University of Singapore, Level 2, Clinical Research Centre Singapore Singapore; ^3^ Community Leadership and Social Innovation Centre (CLASIC) Singapore Institute of Technology Singapore Singapore; ^4^ CSKT Tribal Health St. Ignatius Montana USA; ^5^ Pan American Health Organization/ World Health Organization Office of Equity, Gender, Cultural Diversity Washington District of Columbia USA; ^6^ International Consultant on Ethnicity and Health Pan American Health Organization/ World Health Organization Washington District of Columbia USA

**Keywords:** cultural, ethnic minorities, indigenous peoples, interventions, knowledge, strengths, traditional

The systematic review and meta‐analysis on the international prevalence of prediabetes in children and adolescents from 1996 to 2021 has sparked immense interest in the topic of prediabetes.^1^ The review rendered novel insights into the rapidly rising and worrisome trend of diabetes incidence among young people. Globally, prediabetes prevalence was observed higher in men than in women, in young people aged 10 to 20 years, living in urban regions, and in families with histories of diabetes diagnoses. Diabetes in young persons could potentially lead to disruptive consequences such as early morbidity and a diminished quality of life.^2^ To combat a prediabetes epidemic, the study authors emphasized the need for healthcare professionals to design and implement intensive lifestyle modification interventions. However, differentiated approaches to health are warranted to address the specific needs of indigenous peoples and ethnic minorities in the United States and across Southeast Asia.

A culturally grounded, context‐specific approach for prediabetes wellness and promotion is necessary to attain robust improvements in health outcomes. In particular, indigenous peoples and members of ethnic minorities have been themselves affected by discrimination and negative stereotyping, despite their ecological and cultural richness.^3^ Thus, modern healthcare warrants culturally competent interventions, taking into consideration factors such as ethnicity, culture, and gender.^4^ Diabetes signifies a critical health inequity for indigenous peoples in the United States as the prevalence is more than twice that of any other ethnic groups in the country.^5^ Two out of three indigenous peoples are likely to be diagnosed with kidney failure from uncontrolled diabetes.^5^ Similarly, it is well documented that ethnic minority individuals have higher prevalence of diabetes than nonminority persons.^6^ A recent hermeneutic phenomenological study was conducted to explore the lived experiences of ethnic minority elders with type 2 diabetes mellitus in rural areas of Thailand where health services and support are scarce.^7^ The results highlighted the struggles of ethnic minority elders living with diabetes and the prevalent healthcare inequalities. Healthcare professionals working with ethnic minority individuals in underserved areas should strive to strengthen support systems and services underpinning traditional knowledge and intercultural approach to health.

The provision of prediabetes wellness interventions for indigenous peoples and ethnic minorities in the United States and across Southeast Asia should center strengths of traditional knowledge and approach to health from an intercultural perspective. Studies have found that culture‐specific health interventions underpinning traditional values are vital tenets of health promotion.^8^ While planning, designing, and implementing health promotion interventions for indigenous peoples and ethnic minority communities in the United States and across Southeast Asia, healthcare professionals should achieve culturally and contextually sensitive competencies in (a) examining the intersections of ethnicity with variables such as gender and age, as they render insights on different approaches in which people interact, comprehend, and participate in interventions; (b) understanding representative elements of ethnicity; and (c) acknowledging contextual beliefs and experiences surrounding ethnicity that shape the sustainability of prospective health interventions.^9^

Centering health initiatives surrounding the strengths of traditional knowledge and intercultural approach to health can ensure the interventions are relevant to the needs and preferences of indigenous peoples and ethnic minority communities in the United States and across Southeast Asia. Hence, contextual and cultural sensitivities in readdressing health inequalities can significantly improve outcomes of health within specific communities.

## AUTHOR CONTRIBUTIONS

Sheena Ramazanu was involved in the study conception, design, and writing of the manuscript. Emily Ang, Intan Azura Mokhtar, Jamie Cahoon, Sandra del Pino, and Susana Gomez critically reviewed the manuscript. All authors have contributed significantly and in keeping with the latest guidelines of the International Committee of Medical Journal Editors. The opinions expressed in this manuscript are the responsibility of the authors and do not necessarily reflect the criteria or policy of Pan American Health Organization/World Health Organization.

## CONFLICT OF INTEREST

All authors declare no conflict of interest.
